# Supersensitive Multifluorophore RNA‐FISH for Early Virus Detection and Flow‐FISH by Using Click Chemistry

**DOI:** 10.1002/cbic.202000081

**Published:** 2020-04-20

**Authors:** Nada Raddaoui, Stefano Croce, Florian Geiger, Alexander Borodavka, Leonhard Möckl, Samuele Stazzoni, Bastien Viverge, Christoph Bräuchle, Thomas Frischmuth, Hanna Engelke, Thomas Carell

**Affiliations:** ^1^ Department of Chemistry Ludwig-Maximilians-Universität München Butenandtstraße 5–13 81377 Munich Germany; ^2^ Baseclick GmbH Floriansbogen 2–4 82061 Neuried (München Germany; ^3^ Astbury Centre for Structural Molecular Biology School of Molecular and Cellular Biology, University of Leeds Leeds LS2 9JT UK; ^4^ Department of Biochemistry University of Cambridge Cambridge CB2 1QW UK

**Keywords:** click chemistry, fluorescence probes, mRNA detection, RNA-FISH, viral infection

## Abstract

The reliable detection of transcription events through the quantification of the corresponding mRNA is of paramount importance for the diagnostics of infections and diseases. The quantification and localization analysis of the transcripts of a particular gene allows disease states to be characterized more directly compared to an analysis on the transcriptome wide level. This is particularly needed for the early detection of virus infections as now required for emergent viral diseases, e. g. Covid‐19. *In situ* mRNA analysis, however, is a formidable challenge and currently performed with sets of single‐fluorophore‐containing oligonucleotide probes that hybridize to the mRNA in question. Often a large number of probe strands (>30) are required to get a reliable signal. The more oligonucleotide probes are used, however, the higher the potential off‐target binding effects that create background noise. Here, we used click chemistry and alkyne‐modified DNA oligonucleotides to prepare multiple‐fluorophore‐containing probes. We found that these multiple‐dye probes allow reliable detection and direct visualization of mRNA with only a very small number (5–10) of probe strands. The new method enabled the *in situ* detection of viral transcripts as early as 4 hours after infection.

## Graphical abstract

**More is less**! Increasing the number of fluorophores per oligonucleotide probe allows superb sensitivity, as the fewer the number of probes needed, the lower the background noise. With just a small number of triply labeled strands, RNA‐FISH‐based detection of RNA transcripts was possible in live cells, and a virus could be detected after only 4 hours.

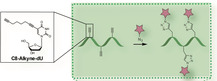

## Introduction

Gene expression varies significantly between individual cells and it is strongly altered in disease states. Viral infections for example lead to early transcription of virus‐specific genes that could be exploited for an early diagnosis and characterization of the infection. In general, basically all malfunctioning processes in cells induce transcriptional changes.^1^, ^2^ These go in hand with altered levels of messenger RNAs (mRNAs). In extreme cases disease related mRNA may not be present at all in the normal state. In most cases however, the levels of specific mRNA will be changed, which requires reliable methods to quantify mRNA transcripts. Detection and quantification of a specific mRNA is thus highly desirable from a diagnostic point of view. Particularly informative are methods that allow the quantification of mRNA levels with spatial resolution. Currently, however, intracellular localization and quantification of mRNA faces a number of challenges that hinder routine use. The most common way to detect mRNA (or other RNAs) in cells is fluorescence *in situ* hybridization (RNA‐FISH).^3^ The method reveals localization patterns of individual RNA transcripts in cells or tissues and as such, RNA‐FISH is the method of choice for quantitative single‐cell transcriptomic studies.^4^, ^5^, ^6^, ^7^, ^8^ The currently available technology behind RNA‐FISH technologies is based on multiple (up to 50) individual anti‐sense single‐stranded (ss) DNA probes, which are approximately 22 nucleotides long. Each probe oligonucleotide carries a single fluorophore, which is typically introduced as its activated NHS ester to an amino group present at the 3’‐end of the probe.^4^, ^5^ The pooled fluorescent ssDNA probes are finally added to fixed and permeabilized cells for hybridization with the target RNA. The large number of probe strands in such experiments is needed to create a sufficiently strong fluorescence signal. However, generally the larger the number of probe oligonucleotides that are used, the larger is often also off‐target staining, which obscures the signal‐to‐noise ratio. A solution to the problem is deconvolution software that is able to increase the specific signal.^9^ From a chemical point of view reduction of the number of probe strands is desirable and this has led to efforts to modify the probe oligonucleotides with, for example, LNA to increase binding. Importantly, mRNA analysis based on flow‐cytometry is so far very challenging with contemporary RNA‐FISH.

Here we report a small FISH‐probe set for mRNA, where every probe contains three fluorophores instead of just one. These multichromophore probes were conveniently prepared using the Cu^I^‐catalyzed azide‐alkyne click reaction.^10^, ^11^, ^12^, ^13^, ^14^, ^15^, ^16^, ^17^, ^18^, ^19^ In order to avoid stacking of the fluorophores on top of each other, which might induce self‐quenching, we chose a fluorophores with two additional sulfonate groups, which provide two negative charges per fluorophore. This is supposed to minimize the interaction with the fluorophores with each other and with the negatively charged DNA. Indeed, with this design a small number of probe strands (5–10) was found to be sufficient for the visualization of RNA transcripts. The new probes design allowed not only transcript quantification and localization by microscopy, but it also enabled transcript analysis using flow‐cytometry.

## Results and Discussion

**Figure 1:**
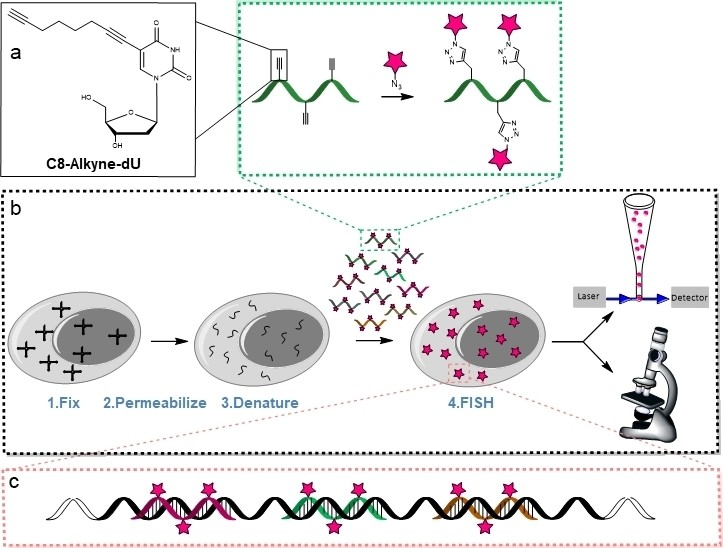
Depiction of RNA‐FISH and schematic representation of the probe synthesis. a) Synthetic oligonucleotides with C8‐alkyne‐dU modifications in various positions were individually conjugated with a fluorescent dye azide. After reaction, the oligonucleotides were mixed to a probe set. b) The probe set was hybridized to the mRNA. After in situ hybridization, the mRNA molecules can be detected by flow‐cytometry and/or microscopy. c) Depiction of the probes labeled with click chemistry hybridized to the target mRNA.

The new procedure based on click chemistry is illustrated in Figure 1. As a test‐system for the mRNA‐FISH we used a HEK293T cell line transfected with a plasmid containing the gene coding for the enhanced green fluorescent protein (eGFP). We synthesized ten DNA probe oligonucleotides targeting specific areas of the eGFP‐mRNA transcript, with each one containing 22 nucleotides (Figure S1 in the Supporting Information). Into each of the ten DNA probe strands we inserted three C8‐alkyne‐dU building blocks at former dT positions (Figure 1 and Table S1) using phosphoramidites that we had developed previously.^20^ The ten triple‐alkyne DNA probes were finally purified by HPLC and individually subjected to a click reaction with Eterneon‐Red 645 azide (cyanine‐5 analogue). The so obtained three Eterneon containing DNA probes (10×3) were finally purified by a simple ethanol precipitation. Due to the high efficiency of the click reaction, no further HPLC purification of the probes was required. For the probes prepared for the virus experiment (*vide infra*) we also confirmed the purity of the obtained probe strand by HPL chromatography (example shown in Figure S2). HPL chromatographic analysis of our 10×3 probe set proved good absorption and fluorescence properties (Figures S3 and S4).

In order to compare the obtained data with the state‐of‐the‐art we performed in parallel studies with a commercially available RNA‐FISH probe set. The provider suggested for the requested detection a set of 30 probe oligonucleotides (Table S2) each one carrying one Quasar 670 fluorophore. The purchased oligonucleotide probes are shown in Figure S1.

In order to investigate the ability to detect RNA, we first performed *in vitro* experiments with isolated total‐RNA obtained from wild‐type HEK293T and HEK293T‐eGFP, stably expressing the eGFP gene. The data are depicted in Figure S5, we obtained clearly visible spots with the (10×3) triple modified probes. Importantly, the 10×3 set provided bright spots even without the use of the special deconvolution software. In order to exclude that the high spot density obtained with the new probes is caused by unspecific binding, we performed a negative control with total RNA isolated from HEK293T cells not expressing the eGFP‐protein (control probes). Here, as expected far fewer spots were obtained, which rules out this possibility.

**Figure 2:**
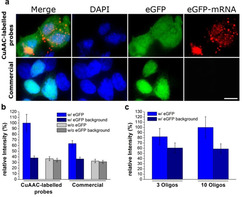
mRNA of eGFP‐expressing HEK cells labeled with 10×3 and with 30×1 probe sets. a) FISH microscopy images of the 10×3 and 30×1 set of probes (scale bar: 10 μm) b) Comparison of the signal and background intensities. c) Signal and background obtained with CuAAC‐labeled probes when using 3 and 10 oligos.

After these *in vitro* experiments, we next investigated the properties of the 10×3 probe set in fixed cells (Figure 2). To this end, the HEK293T cells were grown on 8‐well μ‐Slide (ibidi) and transfected with a plasmid DNA containing a gene coding for eGFP. The cells were fixed and permeabilized using the standard protocols (see Materials in the Supporting Information). We next added the mixture containing our 10×3 probe set and as a positive control, we also performed an experiment with the 30×1 set. Both probe sets were incubated over night at 37 °C. After washing, we analyzed the cells by fluorescence microscopy. The result of the study is shown in Figure 2a. In order to quantify the background fluorescence signal, we calculated the signal intensity obtained after *in situ* hybridization of the probe sets lacking the eGFP‐locus. This background signals for the 10×3 and 30×1 experiment were subtracted from 10×3 and 30×1 data sets obtained with the eGFP expressing cells (Figure 2b). As depicted in Figure 2a we saw for the 10×3 probe set diffraction‐limited spots were detected showing clearly the presence of the complementary mRNAs (Figure 2a, red channel). The signal‐to‐noise ratio was strongly increased. A small caveat is that we noted a slightly increased background signal with the 10×3 probe set (Figure 2b, dark gray bar) likely because the oligonucleotide containing three dyes are more hydrophobic, which may give slightly increased unspecific binding, which increases the background. This effect is obviously overcompensated by the strong increase of the fluorescence signal obtained from specific binding events.

We next experimented with different number of probe strands and dye loading and found that the most reproducible data were indeed obtained with ten oligonucleotide probe strands containing each three fluorophores. In our hands this probe design provided in all investigated cases the best signal‐to‐noise ratios (Figures 2c and S6).

We next investigated if we could further reduce the background signal when we performed the click reaction after *in situ* hybridization as shown in (Figure S7). For this study, we used a set of 10 probe strands with 3 alkyne units, hybridized them with the cellular mRNA and performed the CuAAC‐reaction subsequently *in situ* with a TAMRA azide. While the signal‐to‐noise ratio indeed improved, we noted that we had to perform extensive washing in order to remove additional dye, which is typically used in large excess. This makes this procedure a little more accurate but cumbersome to perform.

**Figure 3:**
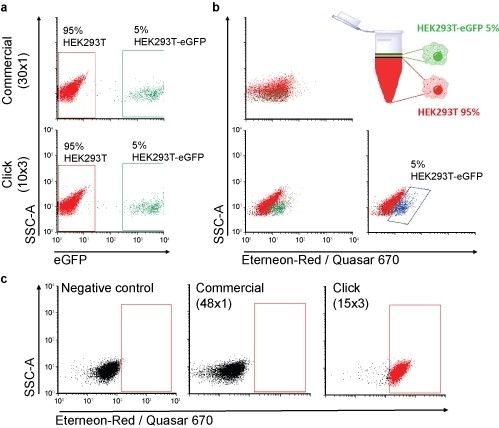
Flow cytometry analysis of mixed HEK293T and HEK293T expressing eGFP in a ratio 20 : 1. a) Mixing ratio determined on the basis of eGFP fluorescence. b) Mixing ratio determined by flow‐FISH. At 0.05 ng/μL of probe, separation was only possible for the 10×3 set and establishes the same ratio when gated, in blue (5 %). c) Flow‐FISH for the detection of the endogenous ABL transcript.

We next investigated if the performance of the 10×3 probe design allows to detect mRNA even using flow‐cytometry in a mixed cell population. For the experiment, we mixed HEK293T cells with and without eGFP‐expression in a ratio 20 : 1 (95 % HEK293T+5 % HEK293T‐eGFP). Then, flow‐cytometric measurements of the mixed cell populations were performed at *λ*_ex_=488 nm/*λ*_em_=530 nm for the detection of the eGFP protein. The correct ratio of the mixed cell population was nicely reproduced (Figure 3a). We then used the flow‐FISH protocol described by Arrigucci et al.,^21^ which involves trypsinization and resuspension of cells. The cells in suspension were permeabilized, fixed and subsequently incubated with the probe sets. Again, we performed the study with the new 10×3 set in comparison to a classical 30×1 design. When we measured at *λ*_ex_=633 nm/*λ*_em_=660 nm, which is suitable for both the Eterneon‐Red 645 azide and the Quasar 670 dye, in the absence of hybridized probes, a single population was observed (Figure S8i). The upper plot of Figure 3b shows the hybridization experiment using the classical probe set at 0.05 ng/μL. Here, only a single population containing both GFP‐positive and GFP‐negative cells was detected at 660 nm. When the 10×3 probe set was used however at the same concentration (lower plot), the GFP‐positive population nicely separated from the GFP‐negative cells. When the separated population was gated (in blue), the exact proportion of cells expressing the eGFP was observed for the two different detection wavelengths. The same result with the classical 30×1 probe set was only obtained, when the concentration was increased fourfold to 0.2 ng DNA/μL (Figure S8ii). These flow‐FISH data show again that the 10×3 probe allows to perform flow‐FISH. Although an exact comparison between the 10×3 and the 30×1 probe sets is not possible, because of the differences in the fluorophore, the connectivity of the fluorophore to the probe and the quality of the strands, we believe that the data support the idea that flow‐FISH is possible with our new probe design that has a limited number of probe oligonucleotides each one carrying three fluorophores.

In order to show that the new probe oligonucleotides are able to report transcription of a relevant endogenous gene by flow‐cytometry, we next tested if the above method would be suitable for the detection of the ABL1‐transcript. This time, we used a slightly larger 15×3 probe set. In order to again obtain comparative information we compared our design with a reported detection that used in this case 48 single labelled oligonucleotides (Semrau et al.).^5^ The results are depicted in Figure 3c. While the 15×3 probe set with only 0.05 ng DNA/μL provides a clear shift in fluorescence compared to the negative control, the 48×1 probe set was under these conditions unable to provide a specific signal separation at this concentration.

We finally explored if the new (10×3) probe design enables imaging of RNA targets that are hard to image with conventional FISH probes. Such targets are characterized by extensive secondary structures that provides only few accessible sites for probe hybridization. We tested our probes by targeting a ∼1 kb RNA viral transcript of the rotavirus A (RVA) gene segment‐7. The idea was to test whether our set of probes detects these transcripts shortly after virus infection when the concentration of the transcript is expected to be very low. This specific target was chosen because of its extensive secondary structure that precludes hybridization of multiple probes, posing additional challenges for conventional FISH.^22^ We fixed rotavirus‐infected cells, 4 hours post infection and incubated them with the (10×3) DNA probe set targeting the RNA regions that were predicted to have less pronounced secondary structures.^23^ To facilitate the detection of virus‐infected cells, we took advantage of a stable cell line that expresses the rotavirus non‐structural protein (NSP5)^24^ fused to eGFP (see Methods in the Supporting Information).

**Figure 4:**
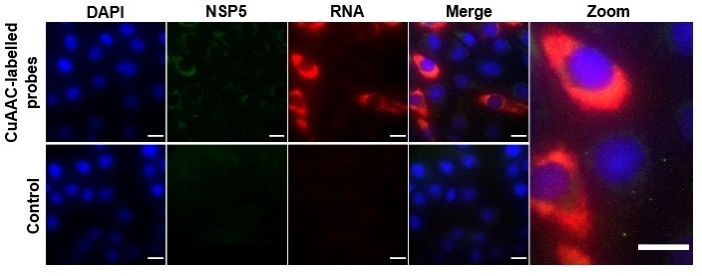
Rotavirus gene segment‐7 RNA transcripts imaged with 10×3 probes. Upper panel: RNA transcripts (red) in virus‐infected cells stably expressing rotavirus non‐structural protein NSP5 fused to eGFP (green) detected 4 hours post infection. Lower panel: Mock‐infected MA104 cells stably expressing NSP5‐eGFP. Scale bars: 20 μm.

After 4 hours post infection RVA, transcripts could be readily detected (Figure 4, top panel, red signal). No transcript specific signal was observed in mock‐infected cells (Figure 4, bottom panel).

In parallel, we also analyzed eGFP‐NSP5‐marked foci in RVA‐infected cells to identify cells at a more advanced stage of infection (Figure 4, top panel, green signal). As expected, these cells showed higher levels of the RNA transcript fully consistent with an increase of the amount of viral RNA transcripts over the course of the viral infection.

Importantly, the increased sensitivity of the new 10×3 detection approach uncovered a number of interesting insights. First, the data show a large variation in the amount of transcripts in different cells at the onset of infection. These variations likely reflect differences in the transcriptional activities of different rotavirus particles.^25^ Furthermore, the transcripts do not seem to accumulate in a particular cellular compartment or in virus‐induced organelles during early infection. These results thus show that the new 10×3 probe design is a significant step forward because it provides new biological insights.

## Conclusions

We show here that by decreasing the number of probes and increasing the number of fluorophores on oligonucleotide probes using click chemistry provides fluorescent probe strands that allow efficient detection of RNA transcripts in live cells. The probes have a superb sensitivity and allow detection of transcripts that due to high secondary structure content provide only a limited number of binding sites. Self‐quenching seems to play only a minor role, possibly because we used fluorophores that carry two negative charges each. The new probe design is so sensitive that it even allows flow RNA‐FISH to be established for demanding flow cytometry applications. These results pave the way for the detection of small highly structure RNA transcripts and transcripts with low abundance. A potential application could be the monitoring of leukemia therapy by flow‐FISH to prevent dangerous relapse cases or the very early detection of virus infections now needed for the detection of the Corona virus to reduce the diagnostic gap and prevent uncontrolled propagation of the disease.

## Conflict of interest

The authors declare no conflict of interest.

## Supporting information

As a service to our authors and readers, this journal provides supporting information supplied by the authors. Such materials are peer reviewed and may be re‐organized for online delivery, but are not copy‐edited or typeset. Technical support issues arising from supporting information (other than missing files) should be addressed to the authors.

Supplementary

## References

[1] A. Raj , A. Van Oudenaarden , Cell 2008, 135, 216–226. 18957198 10.1016/j.cell.2008.09.050 PMC3118044

[2] A. Eldar , M. B. Elowitz , Nature 2010, 467, 167–173. 20829787 10.1038/nature09326 PMC4100692

[3] S. Itzkovitz , A. van Oudenaarden , Nat. Methods 2011, 8, 12–19. 10.1038/nmeth.1573 PMC3158979 21451512

[4] A. Raj , P. van den Bogaard , S. A. Rifkin , A. van Oudenaarden , S. Tyagi , Nat. Methods 2008, 5, 877–879. 18806792 10.1038/nmeth.1253 PMC3126653

[5] A. Raj, S.Tyagi. Detection of individual endogenous RNA transcripts in situ using multiple singly labeled probes. In: Single molecule Tools: Chapter 17, Fluorescence based approaches, Part A; Walter, N. G. B. T.-M. in E., Ed.; Academic Press, **2010**, *472*, 365–368. 10.1016/S0076-6879(10)72004-8 20580972

[6] S. Semrau , N. Crosetto , M. Bienko , M. Boni , P. Bernasconi , Cell Rep. 2011, 6, 18–23. 10.1016/j.celrep.2013.12.002 PMC4214547 24373969

[7] T. Muramoto , D. Cannon , M. Gierliński , A. Corrigan , G. J. Barton , J. R. Chubb , Proc. Mont. Acad. Sci. 2012, 109, 7350–7355. 10.1073/pnas.1117603109 PMC3358836 22529358

[8] A. M. Femino , F. S. Fay , K. Fogarty , R. H. Singer , Science 1998, 280, 585–590. 9554849 10.1126/science.280.5363.585

[9] C. Larsson , I. Grundberg , O. Söderberg , M. Nilsson , Nat. Methods 2010, 7, 395–397. 20383134 10.1038/nmeth.1448

[10] T. Trcek , T. Lionnet , H. Shroff , R. Lehmann , Nat. Protoc. 2017, 12, 1326–1348. 28594816 10.1038/nprot.2017.030 PMC6668020

[11] H. C. Kolb , M. G. Finn , K. B. Sharpless , Angew. Chem. Int. Ed. 2001, 40, 2004–2021; 10.1002/1521-3773(20010601)40:11<2004::AID-ANIE2004>3.0.CO;2-5 11433435

[12] K. V. Gothelf , K. A. Jørgensen , Chem. Rev. 1998, 98, 863–910. 11848917 10.1021/cr970324e

[13] S. Bräse , C. Gil , K. Knepper , V. Zimmermann , Angew. Chem. Int. Ed. 2005, 44, 5188–5240. 10.1002/anie.200400657 16100733

[14] C. W. Tornøe , C. Christensen , M. Meldal , J. Org. Chem. 2002, 67, 3057–3064. 11975567 10.1021/jo011148j

[15] V. V. Rostovtsev , L. G. Green , V. V. Fokin , K. B. Sharpless , Angew. Chem. Int. Ed. 2002, 41, 2596–2599. 10.1002/1521-3773(20020715)41:14<2596::AID-ANIE2596>3.0.CO;2-4 12203546

[16] K. Gutsmiedl , D. Fazio , T. Carell , Chem. Eur. J. 2010, 16, 6877–6883. 20458711 10.1002/chem.201000363

[17] J. Gierlich , G. A. Burley , P. M. E. Gramlich , D. M. Hammond , T. Carell , Org. Lett. 2006, 8, 3639–3642. 16898780 10.1021/ol0610946

[18] P. M. E. Gramlich , S. Warncke , J. Gierlich , T. Carell , Angew. Chem. Int. Ed. 2008, 47, 8350–8358. 10.1002/anie.200705664 18383495

[19] P. M. E. Gramlich , C. T. Wirges , A. Manetto , T. Carell , Angew. Chem. Int. Ed. 2008, 47, 8350–8358. 10.1002/anie.200802077 18814157

[20] S. Hesse , A. Manetto , V. Cassinelli , J. Fuchs , L. Ma , N. Raddaoui , A. Houben , Chromosom. Res. 2016, 24, 299–307. 10.1007/s10577-016-9522-z 27095480

[21] J. Gierlich , G. A. Burley , P. M. E. Gramlich , D. M. Hammond , T. Carell , Org. Lett. 2006, 8, 3639–3642. 16898780 10.1021/ol0610946

[22] R. Arrigucci , Y. Bushkin , F. Radford , K. Lakehal , P. Vir , R. Pine , D. Martin , J. Sugarman , Y. Zhao , G. S. Yap , Nat. Protoc. 2017, 12, 1245–1260. 28518171 10.1038/nprot.2017.039 PMC5548662

[23] A. Borodavka , E. C. Dykeman , W. Schrimpf , D. C. Lamb , elife 2017, 6, e27453. 28922109 10.7554/eLife.27453 PMC5621836

[24] F. Rodriguez , O. R. Burrone , C. Eichwald , J. Gen. Virol. 2004, 85, 625–634. 14993647 10.1099/vir.0.19611-0

[25] E. N. Salgado , S. Upadhyayula , S. C. Harrison , J. Virol. 2017, 91, e00651–17. 28701394 10.1128/JVI.00651-17 PMC5571246

[26] A. Borodavka , U. Desselberger , J. T. Patton , Curr. Opin. Virol. 2018, 33, 106–112. 30145433 10.1016/j.coviro.2018.08.001 PMC6289821

