# Supplementary material for: Supersensitive Multifluorophore RNA‐FISH for Early Virus Detection and Flow‐FISH by Using Click Chemistry
Source: Chembiochem. 2020 Apr 20;21(15):2214–8. doi: 10.1002/cbic.202000081 (PMC7496099; doi:10.1002/cbic.202000081)
Supplement: Supplementary file 1 — Supplementary [file CBIC-21-2214-s001.pdf]

ChemBioChem

Supporting Information

Supersensitive Multifluorophore RNA-FISH for Early Virus Detection and Flow-FISH by Using Click Chemistry

Nada Raddaoui[+], Stefano Croce[+], Florian Geiger, Alexander Borodavka, Leonhard Möckl, Samuele Stazzoni, Bastien Viverge, Christoph Bräuchle, Thomas Frischmuth, Hanna Engelke, and Thomas Carell*© 2020 The Authors. Published by Wiley-VCH Verlag GmbH & Co. KGaA. This is an open access article under the terms of the Creative Commons Attribution License, which permits use, distribution and reproduction in any medium, provided the original work is properly cited.

Eternion Red 645

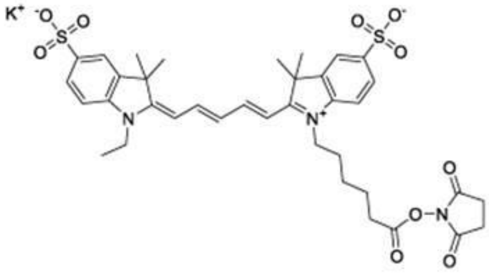

Quasar 670

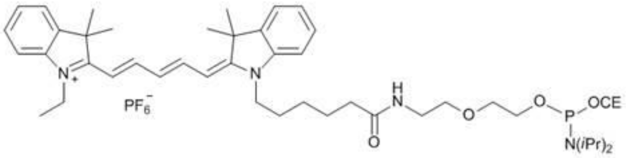

Probe design and sequences for eGFP mRNA

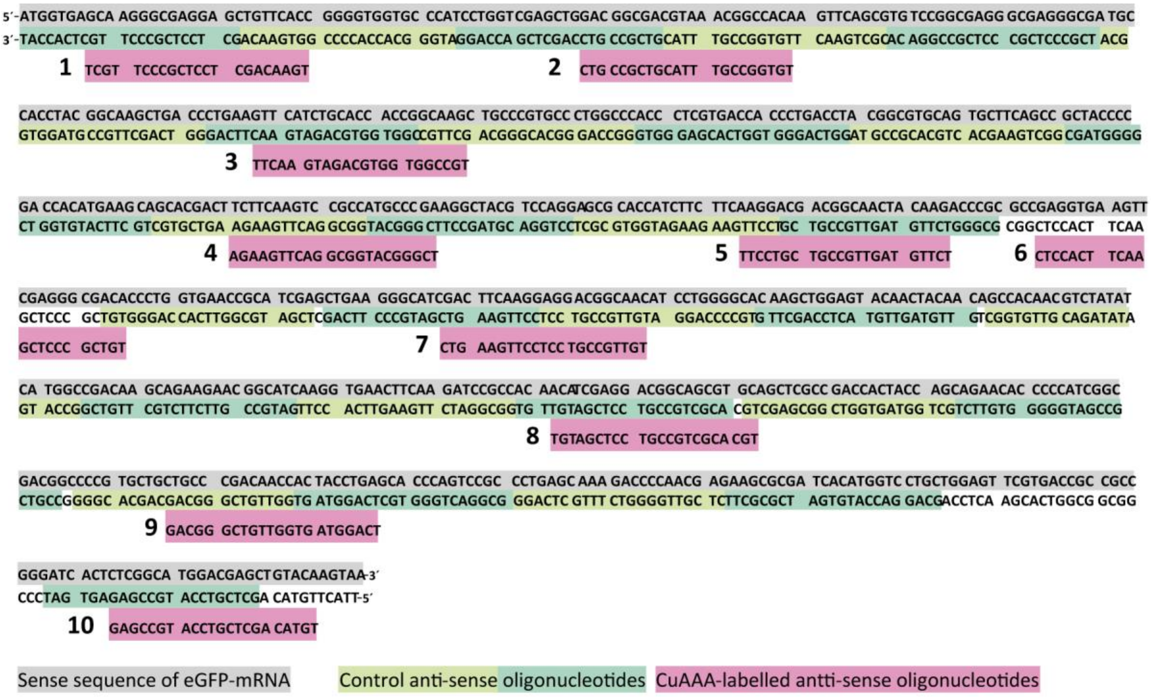

Figure S1. Sequence of the eGFP transcript in grey, together with the sequence of the antisense 10x3 probes in red. The comparison 30x1 probes are shown in green / light green.

Oligonucleotide synthesis

Into each of the DNA strands, three C8-alkyne-dU building blocks were inserted at former dT positions using phosphoramidites developed before[1].

Table S1. Sequences of the 10 oligonucleotide RNA FISH probes and the positions of the C8-alkyne-dU building blocks. The Oligonucleotides listed below were generated for eGFP mRNA using the Stellaris RNA FISH probe designer (https://www.biosearchtech.com/stellaris-designer). The positions of the modifications are highlighted in blue.

Number	Sequence 5'-> 3'
1	TGA ACA GCT CCT CGC CCT TGC T
2	TGT GGC CGT TTA CGT CGC CGT C
3	TGC CGG TGG TGC AGA TGA ACT T
4	TCG GGC ATG GCG GAC TTG AAG A
5	TCT TGT AGT TGC CGT CGT CCT T
6	TGT CGC CCT CGA ACT TCA CCT C
7	TGT TGC CGT CCT CCT TGA AGT C
8	TGC ACG CTG CCG TCC TCG ATG T
9	TCA GGT AGT GGT TGT CGG GCA G
10	TGT ACA GCT CGT CCA TGC CGA G

Click reaction

Each probe was prepared separately. The Cu(I)-catalyzed azide-alkyne cycloaddition (CuAAC) was performed combining 5 nmole of DNA oligo and 45 nmol dye azide: TAMRA-azide (baseclick GmbH) or Eterneon Red 647-azide (baseclick GmbH) in 20 µL reaction volume in the presence of the catalyst CuBr 6.25 mM, tris-hydroxypropyltriazolylmethylamine (THPTA) 12.5 mM, and 2.5 % DMSO. The solution is then incubated for 1 h at 45 °C at 700 RPM protected from light and then cleaned by standard EtOH precipitation before the application in FISH experiments. Further purification was not needed.

HPLC example

For RP-HPLC analysis of labelled probes, a Waters system (Waters e2695 Separation Modul and Waters 2998 PDA) equipped with the XBridgeTM OST C18 column (2.5 µm, 4.6 mm x 50 mm) was used. Linear gradients of buffer A (0.1 M triethylammonium acetate, pH 7.5) to buffer B (0.1 M triethylammonium acetate in 80 % acetonitrile, pH 7.5) were run at a flow rate of 1.5 mL/min at 40 °C column temperature. For detection of oligonucleotide mixtures 260 nm absorbance was chosen.

Method:
Buffer A: 100 % to 70 % in 8 min, 70 % to 15 % in 2 min, 15 % to 0 % in 1 min.

Buffer B: 0 % to 30 % in 8 min, 30 % to 85 % in 2 min, 85 % to 100 % in 1 min

HPL chromatogram example of the oligo before (i) and after click reaction (ii)

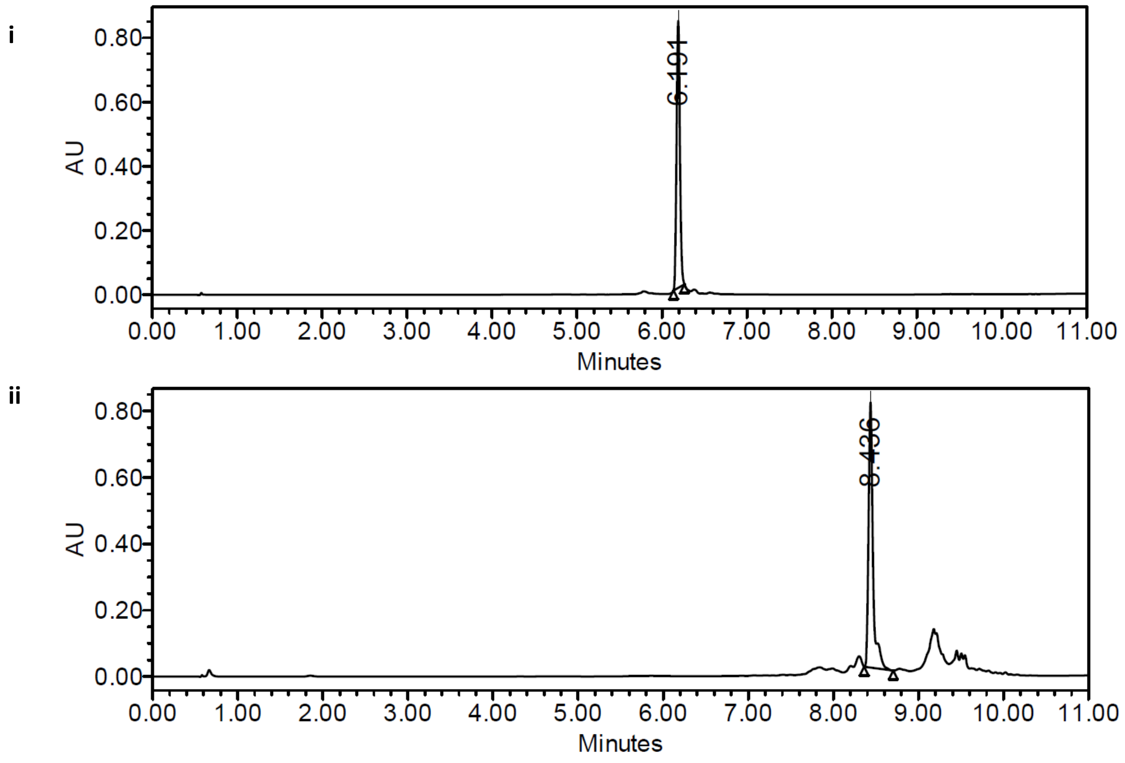

Figure S2. HPLC chromatogram of the probe before (i) and after the click reaction (ii)

Comparison of commercial set of probes and click probes

Analytical RP-HPLC was performed using a Macherey-Nagel Nucleodur 100-3 C18ec column on a Waters Alliance 2695 Separation Module coupled with a 2996 Photodiode Array Detector and a 2475 Multi wavelength Fluorescence Detector, using a flow of 0.5 mL/min. Linear gradients of buffer A (0.1 M triethylammonium acetate, pH 7.0) to buffer B (0.1 M triethylammonium acetate in 80 % acetonitrile, pH 7.0) were run at a flow rate of 0.5 mL/min. For detection of oligonucleotide mixture 260 nm absorbance was chosen, while 647 nm absorbance was used to detect the fluorophore. Method: 0-60 % B in 20 min, 60 to 100 % B in 1 min, 100 % B for 4 min

Table S2. Sequence of the commercial FISH probes, consisting of 30 oligonucleotides with one dye each (30x1)

Number	Sequence 5'-> 3'
1	GCT CCT CGC CCT TGC TCA CCA T
2	ATG GGC ACC ACC CCG GTG AAC A
3	GTC GCC GTC CAG CTC GAC CAG G
4	CGC TGA ACT TGT GGC CGT TTA C
5	TCG CCC TCG CCC TCG CCG GAC A
6	GGT CAG CTT GCC GTA GGT GGC A
7	CGG TGG TGC AGA TGA ACT TCA G
8	GGC CAG GGC ACG GGC AGC TTG C
9	GGT CAG GGT GGT CAC GAG GGT G
10	GGC TGA AGC ACT GCA CGC CGT A
11	TGC TTC ATG TGG TCG GGG TAG C
12	GGC GGA CTT GAA GAA GTC GTG C
13	CCT GGA CGT AGC CTT CGG GCA T
14	TCC TTG AAG AAG ATG GTG CGC T
15	GCG GGT CTT GTA GTT GCC GTC G
16	TCG ATG CGG TTC ACC AGG GTG T
17	CCT TGA AGT CGA TGC CCT TCA G
18	TGC CCC AGG ATG TTG CCG TCC T
19	GTT GTA GTT GTA CTC CAG CTT G
20	GCC ATG ATA TAG ACG TTG TGG C
21	GAT GCC GTT CTT CTG CTT GTC G
22	GGC GGA TCT TGA AGT TCA CCT T
23	ACG CTG CCG TCC TCG ATG TTG T
24	GCT GGT AGT GGT CGG CGA GCT G
25	CCG TCG CCG ATG GGG GTG TTC T
26	GGT TGT CGG GCA GCA GCA CGG G
27	GCG GAC TGG GTG CTC AGG TAG T
28	CTC GTT GGG GTC TTT GCT CAG G
29	GCA GGA CCA TGT GAT CGC GCT T
30	GCT CGT CCA TGC CGA GAG TGA T

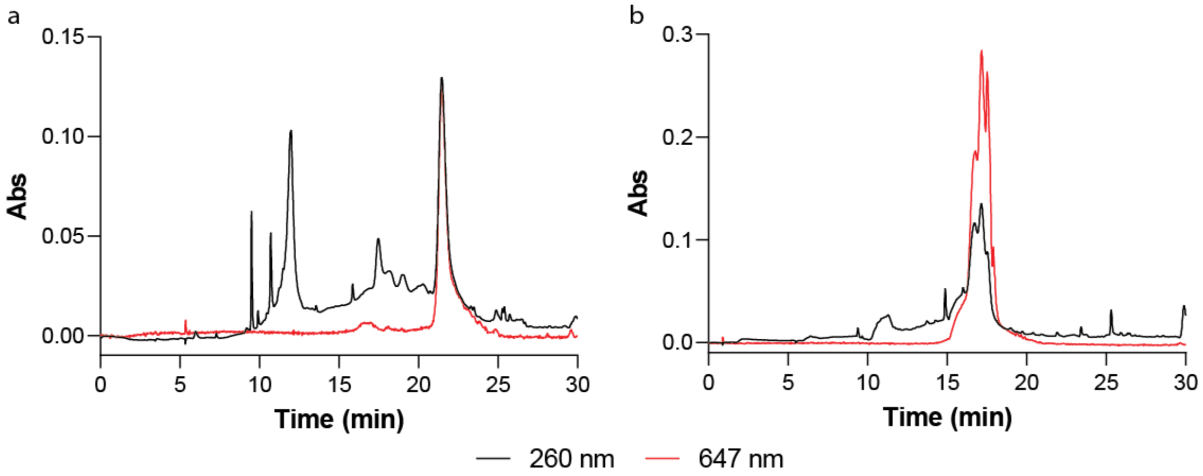

Figure S3. Comparison of (a) a commercial probe set (30x1 Quasar 670 dye) and (b) a clicked probe set (10x3 Eterneon-Red dye). Both sets were loaded in equal amounts, based on 260 nm absorption. Both dyes are very similar cyanine-5 fluorophores.

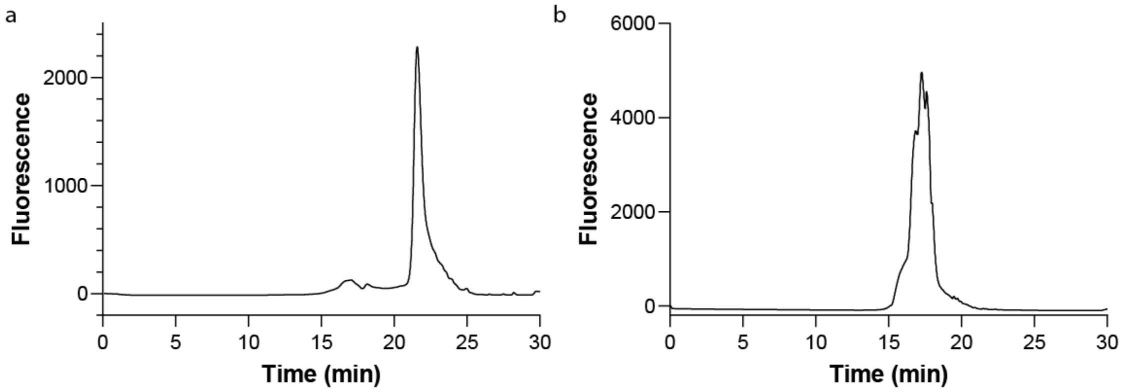

Figure S4. Fluorescence analysis: The fluorescence analysis of the two sets of probes confirm what described previously following the absorbance at 647 nm. Using the same amount of DNA, the commercial probes (a) show an overall lower fluorescence value than the click probes (b). Detection parameters: λ_{exc}: 647 nm, λ_{em}: 665 nm.

In-vitro-FISH with total RNA from eGFP- HEK293T cell line

Total RNA was extracted from eGFP-HEK239T cells using Total-RNA-kit, peqGOLD, catalogue number 732-2868 (VWR). FISH in total RNA was performed as reported in Semrau *et. al.* with minor changes. After DNase digestion, 0.5 µl of total RNA was spotted in the center of an RNase-free cover slips, which was attached to a microscope slide. The total RNA spotted on cover slips were dried for 20 min at 80 °C. After fixation of the spots for 5 min at RT, a washing step with 2x saline sodium citrate (SSC) followed. Either the commercial (30x1, labelled with Quasar 670) or the CuAAC-labelled probes (10x3, Eterneon-Red 647 azide) were added to the spotted RNA in hybridization buffer (300 mM NaCl, 30 mM trisodium citrate, pH 7.0 in nuclease-free water, 10 % (v/v) formamide) and incubated for 5 min at 80 °C. The hybridization buffer including the FISH probes was washed with washing buffer (300 mM NaCl, 30 mM trisodium citrate, pH 7.0, 10 % (v/v) formamide in RNase-free water). The samples were imaged in presence of 2xSSC.

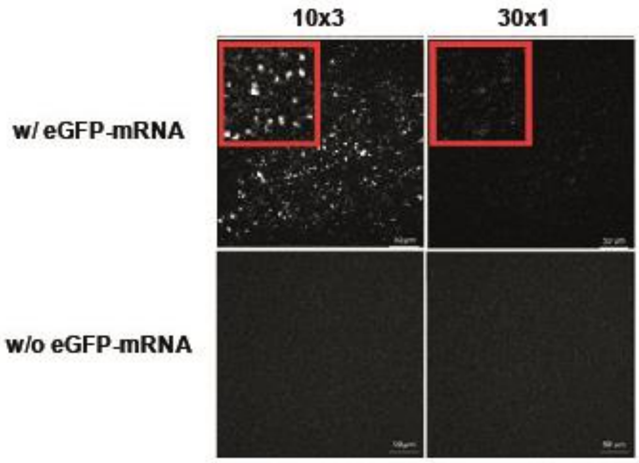

Figure S5. In vitro FISH experiment of total RNA extracted from eGFP-HEK239T cells. Total RNA from eGFP-HEK239T cells hybridized with the CuAAC-labelled probes (10x3) showed a very high signal of diffraction limited spots. In the control experiment, total RNA from eGFP-HEK239T cells was hybridized with the commercial probe set (30x1). Only a very week specific signal was detected. Scale bars: 50 µm

Titration for different numbers of triple labelled oligonucleotides

Since the oligonucleotides were labelled separately via click chemistry, a setting of different numbers of oligonucleotides to a probe set was possible. Four probe sets were prepared: 3x3, 5x3, 7x3 and 10x3 with 10 ng total amount of the oligonucleotides in each set. The sets were hybridized with fixed and permeabilized eGFP-HEK293T and with HEK293T for the negative control without eGFP expression.

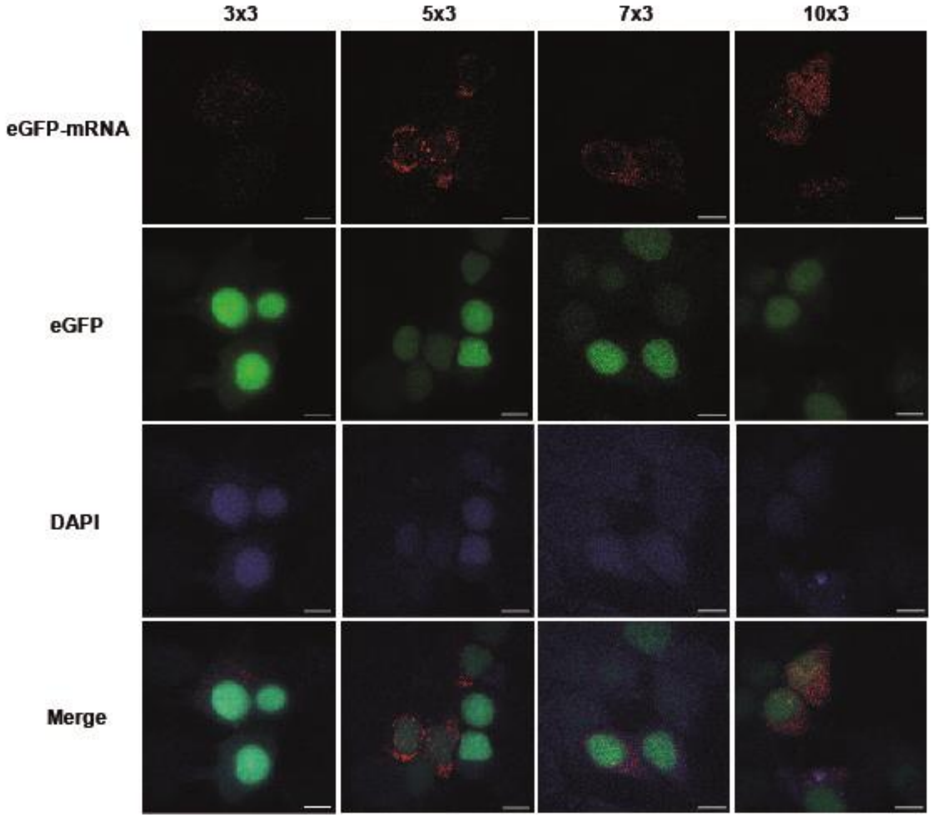

Figure S6. Differences in the signal intensities of eGFP-mRNA using different numbers of triple labelled probes (3x3, 5x3, 7x3 and 10x3) in eGFP-expressing HEK293T cells. Scale bar: 50 µm

Click post hybridization

eGFP-HEK293T were grown in 8 chamber µ-slides cell culture plates (ibidi) until 80 % cell density is reached. After removing the culture medium, cells were washed with 1x RNase-free phosphate saline buffer (PBS) and fixed 15 minutes at RT with the fixation buffer (4 % paraformaldehyde (v/v) in nuclease-free PBS. Cells were then washed twice with 3 % bovine serum albumin (BSA) in 1x PBS and permeabilized with 100 % EtOH for at least 1 h at 4 °C. After permeabilization and removing of the EtOH, cells were washed twice with 3 % BSA in 1x PBS. 5 pmol of each oligonucleotide in 150 µL hybridization buffer (300 mM NaCl, 30 mM trisodium citrate, pH 7.0 in nuclease-free water, 10 % (v/v) formamide) was added to each chamber. The hybridization occurred for 3 h at 37 °C. The unbound oligonucleotides and the hybridization buffer were washed using 10 % washing buffer (300 mM NaCl, 30 mM trisodium citrate, pH 7.0, 10 % (v/v) formamide in RNase-free water) followed by two washing steps with 3 % BSA in 1x PBS. 250 µL of click cocktail ($CuSO_4$ 50 µM, THPTA 250 µM, 7 equivalents 5-TAMRA-PEG3-azide to a final concentration of 25 µM and NaAscorbate to final concentration of 2.5 mM) was added to the cells, which were then incubated 30 minutes at RT protected from light. After hybridization, cells were washed three times either with 3 % BSA (w/v) or DMSO (5, 10, 20, 30, 40 and 50 % in 1x PBS (v/v)). Afterwards, 2x SSC was added to the cells and analysis via microscopy was performed. The signal to noise ratio is reduced when the concentration of DMSO is increased. Scale bar: 50 µm.

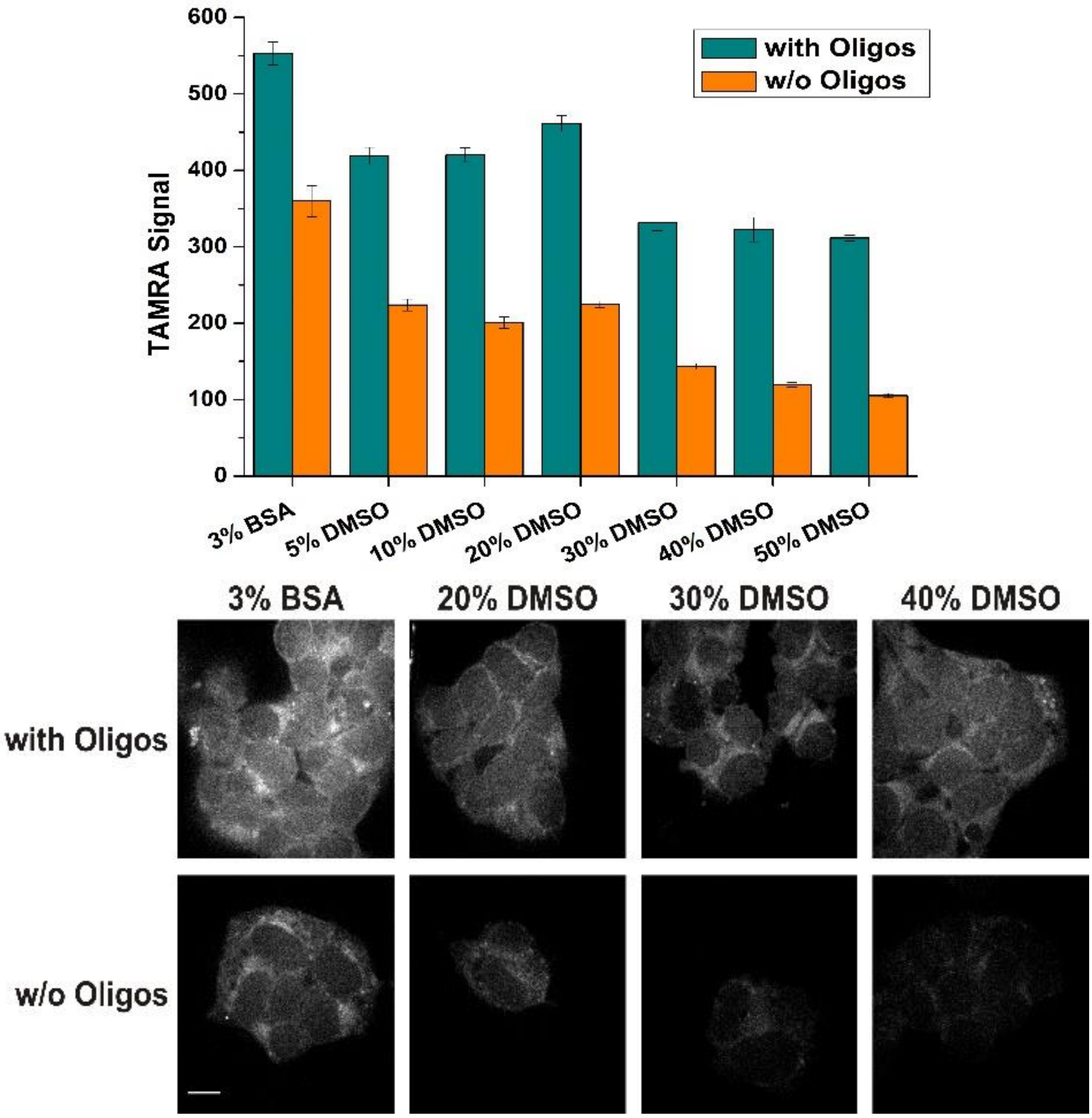

Figure S7. Click reaction of the FISH probes after hybridization.

Cell culture of human cell strains

HEK293T and HEK293T-eGFP cells were cultivated at 37 °C with CO_2-enriched (5 %) atmosphere. Dulbecco's modified Eagle medium (DMEM) supplemented with 10 % fetal bovine serum (FBS), 1 % penicillin and streptomycin, was used as growing medium. When reaching a confluence of 70 % to 80 %, the cells were passaged in a new culture flask. For staining experiments, 1.5×10^4 cells were seeded in each well of a µ-Slide 8 Well from ibidi (ibiTreat, #1.5 polymer coverslip, catalog No. 80826) and cultured for two days or until a density of 80 % is reached.

FISH protocol for HEK293T cells

HEK293T cells were fixed with 4 % (v/v) paraformaldehyde in nuclease-free phosphate saline buffer (PBS) for 10 min at room temperature. Samples were then washed twice with PBS, and the fixed cells were permeabilized with 70 % (v/v) ethanol (200 proof) in RNAse-free water and stored in ethanol at +4 °C for at least 12 hours prior to hybridization. Permeabilized cells were then re-hydrated for 5 min in a pre-hybridization buffer (300 mM NaCl, 30 mM trisodium citrate, pH 7.0 in nuclease-free water, 10 % v/v formamide, supplemented with 2 mM vanadyl ribonucleoside complex). Re-hydrated samples were hybridized with an equimolar mixture of 10 RNA FISH DNA probes specific to bovine rotavirus strain RF gene segment 7 (62.5 nM final concentration, see Table S4) in a total volume of 200 µl of the hybridization buffer (Stellaris RNA FISH hybridization buffer, SMF-HB1-10, Biosearch Technologies, supplemented with 10 % v/v deionized formamide). After 4 hours of incubation at 37 °C in a humidified chamber, samples were briefly rinsed with the wash buffer (300 mM NaCl, 30 mM trisodium citrate, pH 7.0, 10 % v/v formamide in nuclease-free water) after which a fresh aliquot of 300 µl of the wash buffer was applied to each well and incubated twice at 37 °C for 30 min. After washes, nuclei were briefly stained with 300 nM 4',6-diamidino-2-phenylindole (DAPI) solution in 300 mM NaCl, 30 mM trisodium citrate, pH 7.0) and the samples were finally rinsed with and stored in the same buffer without DAPI prior to imaging.

Flow cytometry

FISH experiments for flow cytometry analysis were performed as described previously by Arrigucci et al[2].

Table S3. Sequences of the BCR and ABL FISH probes labelled with click chemistry. The sequences were generated using the Stellaris RNA FISH probe designer (https://www.biosearchtech.com/stellaris-designer). The positions of the C8-Alkyne-dU building blocks are highlighted with blue

Number	Sequence 5'-> 3'
BCR#1	**T**AG CTC T**T**C TTT TCC **T**TG GC
BCR#2	A**T**C CGC **T**CG AAG T**T**G GAC CT
BCR#3	AAC **T**CG ACG T**T**C ACG **T**AG AA
BCR#4	**T**TT TTG CGC **T**CC ATC **T**GC AT
BCR#5	**T**TC AAC **T**CG GCG TCC **T**CG TA
BCR#6	A**T**G CTC **T**GG TAG GGC **T**GG TA
BCR#7	**T**CC GCA A**T**C CTC AAA AC**T** CC
BCR#8	**T**TC TCA T**T**G GAG C**T**G CAG TC
BCR#9	**T**TG TCC CGG AAC A**T**G CGG **T**A
BCR#10	A**T**C TGC G**T**C TCC A**T**G GAA GG
BCR#11	AC**T** CGC TN**T** AGT GGA C**T**C CA
BCR#12	A**T**C TGC **T**GA CTC G**T**C AGC AC
BCR#13	**T**TG TGG A**T**C TCG **T**AG AGC TC
BCR#14	**T**CA GG**T** TCT CGG AGA **T**TT CT
BCR#15	**T**GG CAT C**T**T TGT TGC **T**TC TG
ABL1#1	GCC A**T**T TTT GG**T** TTG GGC **T**T
ABL1#2	**T**TG ACT GGC G**T**G ATG **T**AG TT
ABL1#3	G**T**A ATG G**T**A CAC CC**T** CCC TT
ABL1#4	A**T**G ATG A**T**G AAC CAA C**T**C GG
ABL1#5	A**T**A ATG GAG CG**T** GGT GA**T** GA
ABL1#6	A**T**A GAC AG**T** GGG CT**T** GTT GC
ABL1#7	A**T**C TCC CAC **T**T GTCG **T**AG TT
ABL1#8	**T**TG TGC T**T**C ATG G**T**G ATG TC
ABL1#9	**T**CC TCC T**T**C AAG GTC **T**TC AC
ABL1#10	C**T**T TCA TGA C**T**G CAG C**T**T CT
ABL1#11	AGG **T**TC CCG **T**AG GTC A**T**G AA
ABL1#12	A**T**C TGA G**T**G GCC ATG **T**AC AG
ABL1#13	**T**AC CT**T** CAC CAA G**T**G GTT CT
ABL1#14	A**T**T TGA **T**GG GGA AC**T** TGG CT
ABL1#15	A**T**G GAG AAC **T**TG TTG **T**AG GC

Cells flow cytometer data were obtained using the BD FACS Canto II equipped with air cooled green (488 nm solid state; 20 mW laser output) and red (633 nm HeNe; 17 W output) lasers, and the data were analyzed with the FCS Express 6 (De Novo Software). The fluorescent detectors/filters relevant for this study were FITC (530 +/- 30 nm) and APC (660 +/- 30 nm), respectively. Acquisition was done with a flow rate of 10 µL/min. FSC and SSC were used for detection of forward light scatter parameter and side light scatter parameter. The machine was cleaned before and after each measurement. The photomultiplier tube (PMT) voltage for each parameter was adjusted in order to have the cells displayed in the centre of the investigating plot.

After hybridization and wash, HEK293T or HEK293T-eGFP were immediately analysed by flow cytometer, the samples were maintained in buffer during acquisition.

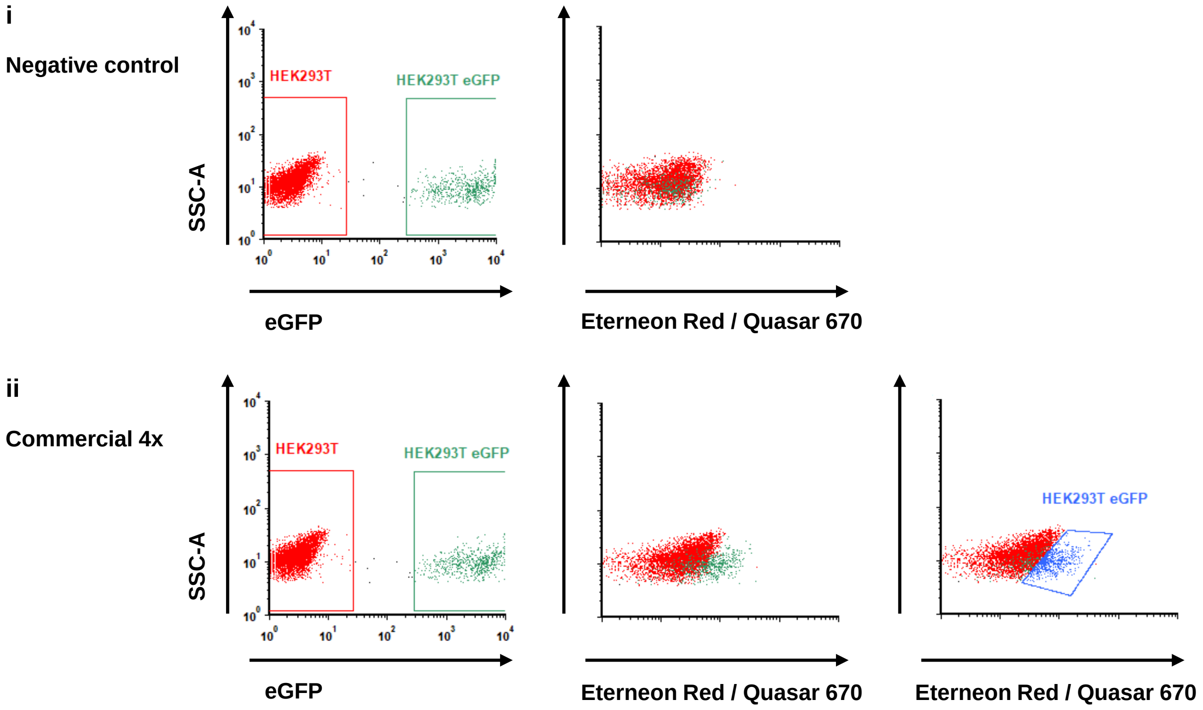

Figure S8. **FACS Dot plot comparison of 30x1 and 10x3 probes.** (i) The negative control of the experiment, where FISH was performed replacing the set of probes with the same volume of H_2O. A population of HEK293T and HEK293TeGFP was mixed in a ratio 20:1, and analyzed by FACS. The eGFP dot-plot reports the correct separation of the HEK293T and HEK293-eGFP. The Eterneon Red dot plot shows a mix of the two populations, as expected. (ii) Same result as the 10x3 probes was obtained with the 30x1 probes set by increasing the concentration four fold to 0.2 ng DNA/µl.

Cells and viruses

The rhesus monkey epithelial cell line MA104 stably expressing NSP5-EGFP [3] was cultured in DMEM (Dulbecco's modified Eagle medium, GlutaMax-I, 4.5 g/L glucose, ThermoFisher), supplemented with 10 % fetal bovine serum (FBS), 1x MEM non-essential amino acids solution (Sigma), 1 mM sodium pyruvate (Sigma) and 500 µg/ml G418 (Roche).
Bovine rotavirus A strain RF (G6P6[1]) was a generous gift from Dr. Ulrich Desselberger (University of Cambridge, UK). It was grown in MA104 Clone 1 cell line (ATCC CRL-2378.1), as described previously [4].
For RNA imaging experiments, MA104 cells expressing NSP5-EGFP were seeded into Ibidi 8-well µ-slides and allowed to reach 90 % confluency prior to the infection. Confluent MA104 cell monolayers were rinsed twice with DMEM medium without FBS for 10 minutes to remove any residual FBS, and were subsequently infected with trypsin-activated bovine rotavirus at multiplicity of infection, MOI = 20.

Rotavirus RNA imaging using RNA FISH

Rotavirus-infected and mock-infected MA104 controls were fixed with 4 % (v/v) paraformaldehyde in nuclease-free phosphate saline buffer (PBS) for 10 min at room temperature. Samples were then washed twice with PBS, and the fixed cells were permeabilized with 70 % (v/v) ethanol (200 proof) in RNAse-free water and stored in ethanol at +4 °C for at least 12 hours prior to hybridization. Permeabilized cells were then re-hydrated for 5 min in a pre-hybridization buffer (300 mM NaCl, 30 mM trisodium citrate, pH 7.0 in nuclease-free water, 10 % v/v formamide, supplemented with 2 mM vanadyl ribonucleoside complex). Re-hydrated samples were hybridized with an equimolar mixture of 10 RNA FISH DNA probes specific to bovine rotavirus strain RF gene segment 7 (62.5 nM final concentration, see SI Table 1) in a total volume of 200 µl of the hybridization buffer (Stellaris RNA FISH hybridization buffer, SMF-HB1-10, Biosearch Technologies, supplemented with 10 % v/v deionized formamide). After 4 hours of incubation at 37 °C in a humidified chamber, samples were briefly rinsed with the wash buffer (300 mM NaCl, 30 mM trisodium citrate, pH 7.0, 10 % v/v formamide in nuclease-free water, after which a fresh aliquot of 300 µl of the wash buffer was applied to each well and incubated twice at 37 °C for 30 min. After washes, nuclei were briefly stained with 300 nM 4',6-diamidino-2-phenylindole (DAPI) solution in 300 mM NaCl, 30 mM trisodium citrate, pH 7.0) and the samples were finally rinsed with and stored in the same buffer without DAPI prior to imaging.

Fluorescent microscopy imaging

HEK cells were imaged on a Zeiss Cell Observer SD microscope equipped with a Yokogawa spinning disk unit CSU-X1 using a 1.40 NA 100x Plan-apochromat oil immersion objective (Zeiss). RNA FISH probes were imaged using 532/561 nm and a BP 690 filter, and eGFP signal was imaged with 488 nm laser excitation and a BP 525/50 filter, respectively. DAPI staining was visualized with 405 nm excitation using a BP 525/50 filter. Data analysis yielding the labeling efficiency was performed using ImageJ.
Rotavirus RNA FISH probes imaging was carried out using a Nikon Ti-E widefield microscope equipped with a 100x 1.40 NA Plan Apochromat oil immersion objective, a CoolLED pE 4000 light source, a Zyla camera and a Quad DAPI/FITC/CY3/CY5 HC filter set.
Z-series spanning the full cell volume with a step size of 0.5 µm at 30 % power with 55 ms exposure times at 385 nm for DAPI, 58 % power with 500-ms exposure times at 470 nm for eGFP and at 35 % power with 500-ms exposures at 550-nm for TAMRA were acquired and the final images represent maximum intensity projections calculated using ImageJ.

Table S4. Into each of the DNA strands designed, three C8-alkyne-dU building blocks were inserted at former dT positions using phosphoramidites developed before[1]. Sequences of the 10 oligonucleotide RNA FISH probes listed below were generated for the rotavirus A gene segment 7 (gene accession no. KF729693.1) using the Stellaris RNA FISH probe designer (https://www.biosearchtech.com/stellaris-designer). The position of the modifications is highlighted in blue.

Number	Sequence 5’ -> 3’
1	ATT GTG GTA TAT TCA ATA CCA
2	AGT CCA TTA TTC TCG TTA TTG
3	GTT TTG CGC ATT TAT TAT GGT
4	AAG GTA TCT TTC CAT TCA GTA
5	GTT CTT GTG TAG AGT CAT ATT
6	ATA GCG TTA TGT CCA TTG GAT
7	ACC AAG TGT TTG TGT ATT TAA
8	TGT AAT CAC TAA CTT CTC CGT
9	CTG TAT GAC TGC TAC GTT CTC
10	GTG GTG TAG TTG TTG GAT CAG

References:

1. Gierlich J, Burley GA, Gramlich PME, Hammond DM, Carell T: **Click Chemistry as a Reliable Method for the High-Density Postsynthetic Functionalization of Alkyne-Modified DNA**. *Org Lett* 2006, **8**:3639–3642.

2. Arrigucci R, Bushkin Y, Radford F, Lakehal K, Vir P, Pine R, Martin D, Sugarman J, Zhao Y, Yap GS, et al.: **FISH-Flow, a protocol for the concurrent detection of mRNA and protein in single cells using fluorescence in situ hybridization and flow cytometry**. *Nat Protoc* 2017, **12**:1245–1260.

3. Eichwald C, Rodriguez JF, Burrone OR: **Characterization of rotavirus NSP2/NSP5 interactions and the dynamics of viroplasm formation**. *J Gen Virol* 2004, **85**:625–634.

4. Cheung W, Gill M, Esposito A, Kaminski CF, Courousse N, Trugnan G, Keshavan N, Desselberger U, Chwetzoff S, Lever A: **Rotaviruses associate with cellular lipid droplet components to replicate in viroplasms, and compounds disrupting or blocking lipid droplets inhibit viroplasm formation and viral replication**. *J Virol* 2010, **84**:6782–6798.
